# Duration of delayed graft function and its impact on graft outcomes in deceased donor kidney transplantation

**DOI:** 10.1186/s12882-022-02777-9

**Published:** 2022-04-19

**Authors:** Pooja Budhiraja, Kunam S Reddy, Richard J Butterfield, Caroline C Jadlowiec, Adyr A. Moss, Hassan A Khamash, Lavanya Kodali, Suman S Misra, Raymond L Heilman

**Affiliations:** 1 Division of Nephrology, Mayo Clinic Hospital, 5777 East Mayo Blvd, Phoenix, AZ 85054 USA; 2 Department of Surgery, Mayo Clinic, 5777 East Mayo Blvd, Phoenix, AZ 85054 USA; 3 Department of Statistics, Mayo Clinic, 5777 East Mayo Blvd, Phoenix, AZ 85054 USA

**Keywords:** Kidney transplant, Deceased donor, Delayed graft function

## Abstract

**Background:**

There is controversy regarding the impact of delayed graft function (DGF) on kidney transplant outcomes. We hypothesize that the duration of DGF, rather than DGF itself, is associated with long-term kidney graft function.

**Methods:**

We analyzed all deceased donor kidney transplants (DDKT) done at our center between 2008 to 2020. We determined factors associated with DGF duration. DGF duration was assessed at three 14-day intervals: < 14 DGF days, 14–27 DGF days, > 28 DGF days.

We studied the impact of DGF duration on survival and graft function and resource utilization, including hospital length of stay and readmissions.

**Results:**

1714 DDKT recipients were included, 59.4% (*n* = 1018) had DGF. The median DGF duration was 10 days IQR (6,15). The majority of recipients (95%) had resolution of DGF within 28 days. Donor factors associated with DGF days were longer cold ischemia time, donor on inotropes, older age, donation after circulatory death, higher terminal creatinine, and hypertension. Recipient factors associated with increased DGF duration included male sex, length on dialysis before transplant, and higher body mass index. There were no differences in acute rejection events or interstitial fibrosis progression by 4 months when comparing DGF days. The median length of stay was 3 days. However, readmissions increased with increasing DGF duration. Death-censored graft survival was not associated with the length of DGF except when DGF lasted > 28 days.

**Conclusions:**

Inferior graft survival was observed only in recipients of DDKT with DGF lasting beyond 28 days. DGF lasting < 28 days had no impact on graft survival. Duration of DGF, rather than DGF itself, is associated with graft survival.

**Trial Registration:**

Retrospective study approved by Mayo Clinic IRB number ID: 20-011561.

**Supplementary Information:**

The online version contains supplementary material available at 10.1186/s12882-022-02777-9.

## Background

The incidence of delayed graft function (DGF) after kidney transplantation has increased substantially over time, as a result of increased utilization of kidneys from high Kidney Donor Profile Index (KDPI) donors, acute kidney injury (AKI) donors, donation after circulatory death (DCD) donors and broader geographic allocation (national sharing) [1, 2]. DGF has historically been associated with inferior graft survival [3–7]. Although high KDPI, AKI, and DCD kidneys are at a higher risk for DGF, there are differences in the kidney allograft survival between the subgroups, with DCD and AKI donors having excellent outcomes [8–10].

DGF has increased resource utilization and concern for poor outcomes, including rejection and worse graft survival [3–7]. However, the data supporting poor outcomes associated with DGF, including rejection and graft survival, remains inconclusive. Some studies suggest decreased graft survival [4, 5], which may be related to a higher rate of rejection [6, 7, 11]; others have not found an association between DGF and acute rejection or graft survival [12]. Delayed graft function is often multifactorial and related to a combination of the donor, transplant, and recipient factors. Other reasons for these differing results may be due to the reporting of DGF as a dichotomous outcome rather than a continuum, different study populations, and center practices.

A few registry-based studies have assessed the impact of DGF duration on graft survival rates [13, 14]. A United Kingdom (UK) registry-based study reported that DGF duration > 14 days [13] was associated with an increased risk of death-censored graft failure. In contrast, an Australian study found a direct time-dependent effect between DGF duration and graft loss [14]. In the United States, although the Scientific Registry of Transplant Recipients database assesses kidney transplant outcomes and reports DGF, it does not provide data specific to DGF days.

We analyzed a large cohort of patients with DGF to determine factors associated with DGF duration as well as the impact of DGF duration on 1) acute rejection, BKV infection, progression of interstitial fibrosis and death-censored graft survival, and 2) resource utilization, including hospital length of stay and readmissions.

## Methods

This is a single-center retrospective study of patients receiving deceased donor kidney transplantation (DDKT) from 2008 to 2020. This study was approved by the Mayo Clinic Institutional Review Board. The last follow-up was at the end of November 2020.

**Fig. 1:**
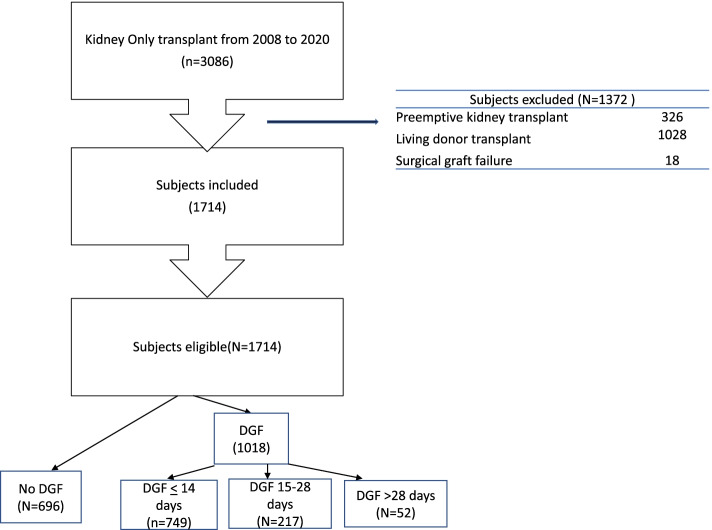
Study diagram

Delayed graft function was defined as the recipient needing dialysis during the first 7 days post-transplant. The last dialysis day was used as the end of DGF. Delayed graft function duration was assessed at three 14-day intervals: < 14 DGF days, 14–27 DGF days, > 28 DGF days. Patients who received multi-organ transplants, preemptive transplants (*n* = 326), living donor kidney transplants (*n* = 1028), and those who had early graft failure within 10 days due to vascular complications were excluded (*n* = 18). Primary nonfunction (PNF) was included in the group with DGF days > 28 and was defined as needing dialysis for > 90 days and no recovery of graft function. The study flow chart is provided in Fig. 1.

Donor AKI was classified per creatinine change as noted in the Acute Kidney Injury Network (AKIN) classification system [15]. At our center, as previously published, we accept kidneys based on kidney biopsy findings [8, 16]. Availability of machine perfusion and pump perfusion parameters do not play a role in decisions regarding kidney utilizations [8, 16]. Donor warm ischemia time was calculated from donor asystole time (time from withdrawal of support to aortic cross-clamp and perfusion with cold preservation fluid).

Protocol post-reperfusion (time-zero) and surveillance biopsies were performed at 4, 12 and 24 months post-transplantation. For-cause biopsies were done in the setting of persistent DGF beyond 2 weeks or for concern for rejection. All rejection episodes were confirmed by biopsy. Subclinical rejection episodes on protocol biopsies were included in the analysis. The biopsies were classified using Banff criteria [17]. We calculated the acute rejection within first 4 and 12 months of kidney transplant, including any for cause and protocol biopsy. Progression of chronic interstitial fibrosis (ci) scoring was done using Banff scores and was defined as an increase in ci score by 1 or more from the baseline biopsy done post-implantation.

BKV infection is diagnosed by BKV viremia, and BKV counts are checked monthly for the first 4 months and then at 6, 8, 12 months, and annually percenters protocol. BKV viremia was defined as detectable BKV viremia, which per our lab assay, is 1600 IU/ml.

All patients received induction therapy. Before 2011, patients received rabbit‐anti thymocyte globulin. After 2011, induction was with alemtuzumab. Patients over age 65 received basiliximab, which did not change during the study period. Patients receiving induction with the depleting agents had complete withdrawal of corticosteroids by post-transplant day 5, while maintenance corticosteroids were continued for those receiving basiliximab induction. Steroids were maintained if recipients had a panel reactive antibody > 80%, donor-specific antibodies, or end-stage renal disease from glomerulonephritis. Maintenance immunosuppression was with tacrolimus and mycophenolate mofetil. Tacrolimus was started on post-transplant days 0–1, irrespective of DGF. Goals for trough tacrolimus levels were 8–10 ng/mL for the first month and then 6–8 ng/mL. Recipients were discharged from the hospital by day 2 to 4, irrespective of DGF, and monitored in the outpatient setting. Patients with ongoing delayed function 2 weeks post-transplant underwent repeat Doppler ultrasound imaging and allograft biopsy.

## Statistical Analysis

Descriptive statistics were reported as mean (standard deviation) or median, interquartile range (IQR) for continuous variables, and frequency (percentage) for categorical variables. We compared donor and recipient-related variables between patients with and without DGF in the entire cohort using the Equal Variance t-test for continuous variables and the Chi-Square test for categorical variables. Nonparametric Kruskal Wallis tests compared data that were heavily skewed.

In the cohort with DGF, the number of DGF days as a continuous variable was modeled using multivariable linear regression with variable inclusion based on univariate significance at *p* < 0.1 level and clinical significance. For analysis of predictors of DGF, on multivariate analysis, KDPI was not included as individual factors of KDPI were not included in Multivariate analysis.

We also divided DGF into 3 groups of 14-day intervals to better characterize the effect of DGF days. We used the above cutoff as previous studies have reported the effect of DGF after 14 days [13]. Here, recipient and donor characteristics were compared by DGF day groups using Analysis of variance (ANOVA) or Chi-Sq, where appropriate. We tested different cutoff points for the duration of DGF using the Contal and O'Quigley method [18]. Primary nonfunction were not included in the Contal and O'Quigley calculation.

Our primary outcome was to study the effect of DGF days on death censored graft survival. We used the unadjusted Kaplan Meier method to estimate death censored graft survival between 14-day groups of DGF days. We performed Kaplan Meier with and without PNF cases.

We used the adjusted Cox Proportional Hazard model when considering DGF days as a continuous variable in the subgroup of patients with DGF for death censored Graft survival analysis.

We also studied effect of DGF vs. no DGF and between 14-day groups of DGF days on patient survival using unadjusted Kaplan Meier. We also reported causes of death and death censored graft survival in the no DGF and DGF subgroups.

We compared the incidence of acute rejection, the occurrence of BKV infection, progression of interstitial fibrosis (from preimplantation to 4 months), length of stay, and readmissions at 30 and 90-days post-transplant in the DGF groups. Readmission rates included observation status and inpatient stay > 24 h. We don't admit subjects for renal biopsy or outpatient procedures.

In case of missing data, we excluded the missing data from the numerator and denominator. Imputation for missing data was not performed.

All statistical analyses were two-sided and considered statistically significant at the *p* = 0.05 level. Analyses were performed in SAS v9.4 (SAS Institute; Cary, NC).

## Results

There were 1018 (59%) recipients with DGF and 696 (41%) without DGF. The majority of patients had resolution of DGF by 14 days (*n* = 749, 74%), while 21% (*n* = 217) had resolution of DGF between 15–28 days and 5% (*n* = 52) had DGF lasting > 28 days. The median duration of dialysis days in the DGF group was 10 [6, 15]. The median duration of dialysis days (IQR) was 34.5 days (31,39.75) in the DGF group needing dialysis for > 28 days, after excluding 7 PNF.

Baseline recipient and donor characteristics are shown in (Table 1). With an increasing number of DGF days, it was more common to see kidney allografts coming from donors with hypertension, a higher KDPI score, DCD status, and AKI. Similarly, an increase in cold ischemia time (CIT) was associated with a longer DGF duration. (Table 1).

**Table 1:** Comparison of Groups Based on Delayed Graft Function (DGF) Days

	**No DGF**	**DGF days**			***P* value**
	0 (*n* = 696)	< 14 (*n* = 749)	15–28 (*n* = 217)	> 28 (*n* = 52)	
Donor age (years)	36.9 (19.1)	40(15.6)	41.5(14.8)	46(12)	< 0.001
Male	425 (61%)	463(62%)	146(66.4%)	19 (47.5%)	0.135
Donor Body mass index (kg/m^2^)	26.7 (7.4)	29.9(7.8)	29.6(7.6)	30(10.5)	< 0.001
Donor Hypertension	161(24.2%)	209 (29%)	62 (29%)	22 (46%)	0.005
Donor Black race	46 (6.6%)	62(8%)	15(7%)	2(4%)	0.47
Donor Acute Kidney Injury stage ≥ 2	94 (13.5%)	385(51%)	130(60%)	20(39%)	< 0.001
KDPI	46.6 (28.9)	52.7(24.7)	54.8(25.1)	62 [21]	< 0.001
KDPI ≥ 85	104 (15%)	92(12%)	32(15%)	9(17%)	0.5
Donation after circulatory death	106(15.2%)	196(26%)	59(27%)	20(39%)	< 0.001
Donor on inotropes	213(30.6%)	243(32%)	78(36%)	15(38%)	0.43
Donor Diabetes mellitus	69(10.1%)	57(7.8%)	16(7.4%)	6(12%)	0.31
Donor terminal creatinine (mg/dl)	1.27(1.39)	3.1(2.8)	3.6(2.9)	2.5(2.3)	< 0.001
Cold Ischemia time > 24 h	125(18%)	247(33%)	68(31%)	22(42%)	< 0.001
Cold Ischemia time (hours)	16.9 (7.3)	20.8(6.7)	21.4(6.2)	21.7[8]	< 0.001
Pumped kidney	60(9%)	92(12%)	21(10%)	8 (15%)	0.4
Warm ischemia time (minutes)	28.11[12]	24.8[10]	29 [11]	21 (6.8)	0.32
Recipient Male	351(50.4%)	482(64%)	145(66%)	32(76%)	< 0.001
Recipient Black race	68(9.8%)	93(12.4%)	31(14.3%)	5(9.6%)	0.21
Recipient Age at transplant (years)	54.4(14.0)	56.1[13]	56.5[12]	56.8[12]	0.04
Recipient Body mass index (kg/m^2^)	28.1(5.6)	29.1(5.6)	29.9(5.8)	31.2(5.5)	< 0.001
Recipient Length on Dialysis (days)	1306.2(1042)	1365.3(942)	1504(895)	1540 (850)	0.04
Pretransplant Diabetes mellitus	250(35.9%)	347(46%)	120(55%)	28(54%)	< 0.001
Previous transplant	87(12.5%)	69(9.2%)	21(9.5%)	5(9.6%)	0.21
Panel reactive antibody (%)	21.91(35.5)	17.8(32.4)	18.7(31.6)	16.5(29.5)	0.11
Induction Basiliximab Rabbit‐anti thymocyte globulin Alemtuzumab	Basiliximab-165(25%) Rabbit‐anti thymocyte globulin -139(21%) Alemtuzumab-355(54%)	Basiliximab-203 (29%) Rabbit‐anti thymocyte globulin -87 (12%) Alemtuzumab-418(59%)	Basiliximab-60(29%) Rabbit‐anti thymocyte globulin -26(13%) Alemtuzumab-120(59%)	Basiliximab-15(33%) Rabbit‐anti thymocyte globulin -6(13%) Alemtuzumab-26 (54%)	< 0.001
Continuous variables given as mean (standard deviation); categorical variables given as frequency (percentage)					

## Death censored graft and patient survival

**Fig. 2:**
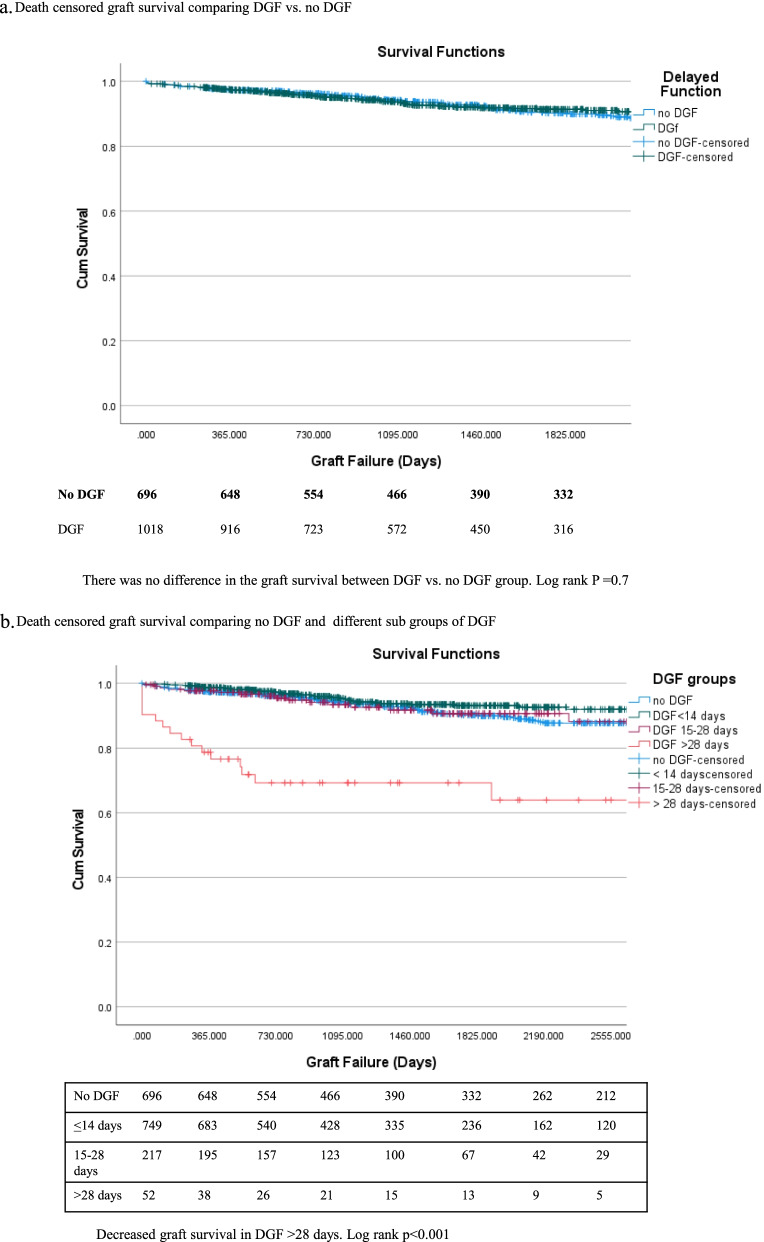
**a** Death censored graft survival comparing DGF vs. no DGF. **b** Death censored graft survival comparing no DGF and different sub groups of DGF

Overall, death-censored graft survival (log-rank *P*—value = 0.302) was similar between the DGF and no DGF groups ((Fig.2a), Log-rank *p* = 0.57). When we tested different cutoff points for the duration of DGF using the Contal and O'Quigley Method, 28 days of DGF was identified as the significant cutoff point where the hazard ratio was 3.813 (*p* < 0.001). When we used 7 days cut-off, it was not significant. However, when we ran the model excluding the hyperkalemia (DGF days = 1), this subgroup upheld findings.

Kaplan–Meier graft survival in the 3 subgroups of DGF compared to no DGF groups is shown in (Fig.2b). Delayed graft function > 28 days (log-rank *p* < 0.001) was associated with inferior graft survival. After excluding PNF, DGF > 28 days was associated with increased death censored graft survival (log-rank *p* < 0.001).

On multivariate analysis, Cox proportional hazard model DGF > 28 days was associated with higher death censored graft failure [2.85 (1.3–6.1), *P* = 0.008]. (Table 2) Delayed graft function duration, when used as a continuous variable for the group with < 28 days of DGF, it was not associated with graft loss (*p* = 0.783). (Table 2) An increase in KDPI score was associated with increased hazards of graft loss{1.013 (1.01–1.02), *p* = 0.001}. (Table 2).

**Table 2:** Death Censored Graft Survival

		**Univariate**		**Multivariable**	
**Variable**	**Level**	**HR (95% CI)**	***P*-value**	**HR (95% CI)**	***P*-value**
Delayed function	Yes vs. No	0.83 (0.59, 1.12)	0.30		
DGF (more than or less than 28 days)	> 28 days vs ≤ 28 days	3.3 (1.55–7.14)	0.002	2.85 (1.3–6.1)	0.008
Days of DGF for those with DGF < / = 28 days	One day Increase	0.99(0.97–1.02)	0.783		
Age at Transplant	One Unit Increase	0.99 (0.97, 1.004)	0.16		
Recipient Sex	Male vs. female	1.12 (0.79, 1.16)	0.59		
Recipient Race	Black vs. White	1.56 (0.9, 2.5)	0.055	1.61(1.01, 2.58)	0.05
Recipient Body mass index (kg/m^2^)	One Unit Increase	1.01 (0.98, 1.04)	0.66		
Recipient pre transplant Diabetes mellitus	Yes vs. No	1.13(0.82, 1.56)	0.46		
Previous Kidney Transplant	Yes vs. No	1.093 (0.66, 1.82)	0.73		
Length Dialysis (days)	One Unit Increase	1.002 (0.997, 1.01)	0.39		
Donor Acute Kidney Injury (> / = 2)	Yes vs. No	0.94 (0.67, 1.33)	0.74		
Kidney Donor Profile Index	One Unit Increase	1.01 (1.002, 1.015)	0.01	1.013 (1.01–1.02)	0.001
Cold Ischemia Time (hours)	One Unit Increase	1.013 (0.99, 1.04)	0.29		
Warm Ischemia Time (minutes)	One Unit Increase	1.02(0.96,1.09)	0.55		
Panel of Reactive Antibody (%)	One Unit Increase	1.00(0.99,1.01)	0.59		
Histocompatibility Antigen mismatches	One increase	1.09(0.97,1.22)	0.15		

**Fig. 3:**
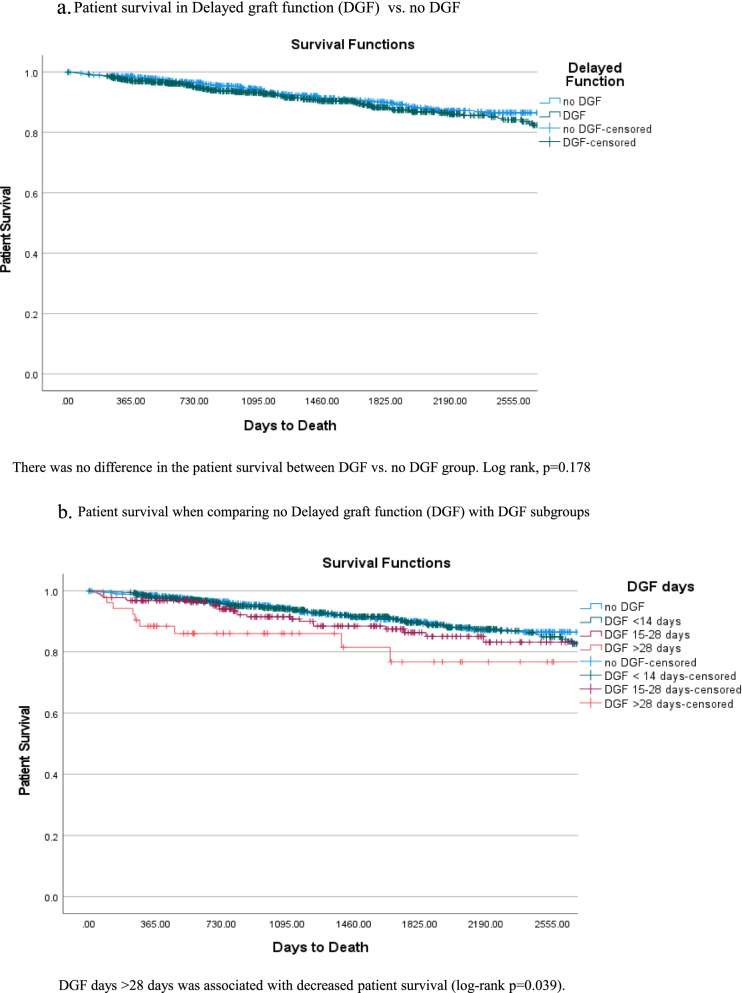
**a** Patient survival in Delayed graft function (DGF) vs. no DGF. **b** Patient survival when comparing no Delayed graft function (DGF) with DGF subgroups

There was no difference in patient survival between DGF vs. no DGF group (log-rank *p* = 0.178) (Fig.3a). However, DGF days > 28 days was associated with decreased patient survival (log-rank *p* = 0.039) (Fig.3b).

There was higher death in 1 year in the subjects with DGF > 28days (1.5% in no DGF, 2.1% in DGF < 14 days, 3.2% in DGF 15–28days, and 11.5% in DGF > 28 days, *p* < 0.001). The cause of death in the group with DGF days > 28 days was an infection. Causes of death censored graft loss and death within 1year are provided in Supplement (Tables 1 and 2).

## Acute rejection and BKV infection

The rate of acute rejection, including subclinical rejection within first 4 months ater transplant. Rate of acute rejection including subclinical rejection within 12 months was 19.5% in no DGF, 13.4% in DGF < 14 days, 19.5% in DGF 15–28 days and higher at 23% in DGF > 28 days (*p* = 0.005).

Rate of BK infection within the first year, was similar between the groups (Table 3).

**Table 3:** Acute Rejection, BKV, Length of Stay, Readmissions and Pathology

	**No DGF**	**DGF days**			
	**0 (*n* = 696)**	** < 14 (*n* = 749)**	**15–28 (*n* = 217)**	** > 28 (*n* = 52)**	***P* value**
Acute rejection including subclinical rejection within 4 months of transplant	79(11.4%)	68(9.1%)	25(11.4%)	10(19%)	0.09
Acute rejection including subclinical rejection within 12 months of transplant	136(19.5%)	100(13.4%)	43(19.5%)	12(23.1%)	0.005
BKV infection within 1 year of transplant	116(16.7%)	106(14.2%)	31(14.3%)	4 (7.7%)	0.24
Length of stay (days) (Median)	3(2,4)	3(2,4)	3(2,4)	3(2,5)	
Readmission at 30 days	178(26%)	266(36%)	108(50%)	25(48%)	< 0.001
Readmission at 90 days	239(34%)	346(46%)	132(61%)	32(64%)	< 0.001
Pathology	0 (*n* = 696)	< 14 (*n* = 749)	15–28 (*n* = 217)	> 28 (*n* = 52)	
Ci progression at 4 month (+ 2)					
Missing	372	334	108	36	
Yes	154	200	64	14	0.196
No	170	215	45	12	
c**i** score at time 0 (time 0 ci score)					< 0.001
Missing	274(39%)	223(30%)	67(30%)	21(40%)	
ci0	342(49%)	393(53%)	108(50%)	20(39%)	
ci1	77(11%)	126(17%)	41(19%)	10(20%)	
ci > 1	3 (0.4%)	7(0.9%)	1(0.5%)	1(2%)	
C**i** score at 4 month					< 0.001
missing	199 (29%)	179(24%)	61(29%)	21(40%)*	
c0	231(33%)	200(27%)	40(18%)	8(15%)	
c1	228(33%)	314(42%)	95 (43%)	12(23%)	
ci > 1	38(5%)	56(8%)	21(10%)	11(21%)	

Ci chronic interstitial fibrosis, *ci at 4 months was not available in 40% of subjects with DGF > 28 days. Continuous variables given as mean (standard deviation); categorical variables given as number (percentage)

## Chronic interstitial fibrosis progression

Chronic interstitial fibrosis (ci) progression at 4 months was similar in the no DGF and DGF groups (*p* = 0.45) (Table 3). Delayed graft function days (*p* = 0.21) were not associated with ci progression at 4 months on logistic regression. Table 3 shows ci scores at times 0 and 4 months. In the group with DGF > 28 days at 4 months, only 60% had protocol biopsy at 4 months compared to 71–76% in other groups. The reasons for 40% (*n* = 21) not getting protocol biopsy in DGF > 28 days included: 7 had graft loss, 2 died, 2 were decapsulated kidneys, 1was on anti-coagulation, 1 had a recent infection, and 8 were not done as they had biopsy around one month for evaluation of DGF and had stable graft function.

## Length of stay and readmission in 30 and 90 days (Table 3)

The median length of stay was 3 days for all the groups. Readmissions within 30 days were 26% in no DGF, 36% in DGF ≤ 14 days and around 50% for DGF > 14 days (Table 3). Readmissions in 90 days increased with the duration of DGF. Thirty-four percent in no DGF group, 46% in DGF < 14 days, 61% is DGF 15–28 days and 64% in DGF lasting > 28 days (*p* < 0.001).

## Risk factors for DGF (Table 4)

**Table 4:** Predictors of DGF Days

	**Univariate**		**Multivariable**	
**Variable**	**Estimate (SE)**	***P*** **-value**	**Estimate (SE)**	***P*** **-value**
Recipient Age at Transplant	0.05 (0.02)	0.02	-0.19(0.02)	0.3
Recipient sex (male vs female)	2.3(0.4)	< 0.001	1.7 (0.4)	< .001
Recipient Race (Black vs White)	0.82(0.9)	0.34		
Recipient body mass index(kg/m^2^)	0.24 (0.05)	< 0.001	0.11 (0.04)	0.004
Diabetes Pretransplant Diabetes mellitus	2.6(0.54)	< .0001	0.64 (0.4)	0.15
Previous Kidney Transplant	-0.366 (0.8)	0.68		
Length Dialysis (days)	0.02 (0.01)	0.02	0.001 (0.01)	0.02
Donor Acute Kidney Injury ≥ 2	5.23 (0.376)	< .0001		
Kidney Donor Profile Index	0.05 (0.01)	< 0.001		
Cold ischemia Time (hours)	0.3 (0.04)	< 0.001	0.19 (0.03)	< .001
Donor Hypertension	2.1(0.6) (0.437)	0.001	1.3(0.5)	0.01
Donor Oliguria/Anuria	5.0(0.5)	< .001		
Donor age	0.06 (0.02)	< .001	0.03 (0.01)	0.03
Donor Sex (male vs female)	0.12 (0.4)	0.7		
Donor Race (Black vs. White)	-0.2(1.4)	0.88	-1.5(0.8)	0.08
Donor body mass index (kg/m^2^)	0.16(0.04)	< 0.001	0.02(0.03)	0.48
Donation after Circulatory Death	2.8(0.6)	< .001	3.3 (0.5)	< .001
Donor Diabetes Mellitus	-0.765 (0.68)	0.26		
Panel Reactive Antibody (%)	-0.01(0.1)	0.23		
Warm Ischemia Time (minutes)	-0.08(0.08)	0.29		
Donor on inotropes	1.05(0.4)	0.02	1.2(0.4)	0.01
Terminal donor creatinine (mg/dl)	0.98(0.1)	< .0001	0.96(0.08)	< 0.001

Multivariate analysis of donor and recipient factors associated with the duration of DGF days is shown in Table 4. Donor factors associated with DGF duration were longer CIT (0.19), donor on inotropes (1.2), older age (0.03), donation after circulatory death (3.3), higher terminal creatinine (0.96), and hypertension (1.3). Recipient factors associated with DGF duration included male sex (1.7), pretransplant dialysis days (0.001), and higher body mass index (0.11).

## Subgroup analysis of DGF duration > 28 Days

On univariate analysis, a higher KDPI score [62(21) vs. 53.2(25), *p* = 0.013] and donor HTN (44% vs. 29%, *p* = 0.04) were more commonly observed in patients with DGF lasting > 28 days compared to ≤ 28 days (Table 3 for supplement). Post-transplant recipient factors contributing to prolonged DGF included infection (*n* = 6), cardiovascular-related complications (*n* = 7), cirrhosis-related decompensation (*n* = 1), acute rejection (*n* = 2), reoccurrence of focal segmental glomerulosclerosis (*n* = 2), hemolytic uremic syndrome (*n* = 1), fibrin thrombi and later sepsis (*n* = 1) and post-transplant bleeding (*n* = 3).

Ten patients needed dialysis for 90 or more days. Of these 7 were declared PNF: 1 recipient received a standard KDPI kidney and had acute rejection and pyelonephritis; 1 recipient received an AKI and high KDPI kidney; 2 recipients had chronic hypotension due to cardiac causes; 1 recipient had hypotension due to cirrhosis; 2 recipients had graft loss likely related to advanced chronic changes on time-0 biopsy (ah1, cg0, ci2-3, ct2-3, cv2-3); and a seventh recipient received an AKI kidney and had hypotension and focal segmental glomerulosclerosis recurrence post-transplant.

Three recipients came off dialysis after being dialyzed for 90 days. One needed dialysis for 6 months due to post-transplant thrombotic microangiopathy with cortical necrosis but came off dialysis and has had satisfactory graft function for more than 4 years. This patient received a kidney from a young donor with a KDPI score < 20%. Two subjects had chronic changes on biopsy and maintained graft function after being switched to belatacept.

## Discussion

The true impact of DGF on kidney transplant outcomes remains debated, and in most studies, DGF is reported as a dichotomous outcome rather than a continuum. During this study period, we assessed 1714 DDKT of which, 59% (*n* = 1018) had DGF. We observed a median DGF duration of 10 days, with the majority of recipients (95%) showing resolution of DGF within 28 days. There were no differences in acute rejection events or interstitial fibrosis progression by 4 months when comparing DGF days. Readmissions increased with increasing DGF duration. Death-censored graft survival was not associated with the length of DGF except when DGF lasted > 28 days. To our knowledge, this is the first study to examine in detail the effect of DGF duration on rejection, readmissions, graft survival, and histology using a large cohort of deceased donor kidneys with DGF.

In a UK single center [7] study with DCD donors from 2011 to 2016, the presence of DGF was associated with lower graft survival, though the duration of DGF was not. In contrast, a UK registry-based DCD study [13] reported that DGF > 14 days was associated with an increased risk of death-censored graft failure (hazard ratio 1·7, *p* = ·001) and recipient death (hazard ratio 1·8, *p* < 001) compared to grafts with immediate function [13]. That study reported a 2.5 times higher incidence of acute rejection within 3 months in recipients with DGF lasting > 14 days than those with DGF duration < 7 days. Because this was a registry-based study, there was insufficient data on induction and maintenance immunosuppression and if transplant centers held tacrolimus in the setting of DGF.

Authors Lim et al., using the Australian and New Zealand Dialysis and Transplant Registry, reported a direct effect between DGF duration and death-censored graft loss. [14] The authors reported DGF > 7 days was associated with a greater than 40% risk of graft loss. The Hazard Ratio for Death censored graft loss for DGF duration 8–13 days and > 14 days was 1.45 (1–2.1) and 1.6 (1.1–2.3) when compared to DGF duration 1–4 days. Suggesting, DGF duration > 7 days had a 45% higher relative risk for Death censored graft loss over the entire follow-up period.

The authors also reported an association between DGF duration and risk for acute rejection [1.17 (1.10–1.25; *p* < 0.001)]; subjects who developed acute rejection at 6 months were more likely to have graft loss [14]. Although this reported association is worrisome, it is important to note that the study had a higher incidence of acute rejection, 30% risk at 6 months, greater than expected. The lower use of T-cell depleting induction (3.5% with DGF vs. 10.7% without DGF) may have contributed to this finding [14]. In our present study, DGF days did not negatively impact death-censored graft survival except for those patients with DGF duration > 28 days. Duration of DGF also had no impact on acute rejection as compared to the registry-based studies mentioned above. By comparison, depleting agents were used in 70% of our recipients with DGF. By protocol, our center also does not modify induction or delay initiation of calcineurin inhibitors in the setting of DGF. Our center's practice of early tacrolimus initiation combined with higher levels (8–10 ng/ml) within the first month of the transplant could be reasons for these observed differences in early rejection.

There is controversy if an increase in Cold Ischemia Time and DCD kidneys is associated with an increased risk of BKV replication due to ischemia–reperfusion causing viral activation [21–23]. We did not find an association between DGF and BKV infection in this cohort.

Despite some data suggesting otherwise, there continue to be concerns in the transplant community regarding the impact of DGF on the progression of allograft interstitial fibrosis. We have previously demonstrated that DGF does not increase the risk for interstitial fibrosis at one year [19]. In the current study, we studied the effect of DGF duration on the risk of interstitial fibrosis progression. We did not find any significant impact of DGF days on the progression of chronic interstitial fibrosis compared to time 0 post-reperfusion biopsies to 4 months protocol.

Our center aims to discharge patients on post-transplant days 2–3 irrespective of DGF. As we have previously reported, our center's protocol is to routinely discharge patients with outpatient non-hospital based hemodialysis and close follow-up in our outpatient transplant clinic to minimize hospital length of stay. The median length of stay was 3 days, irrespective of DGF days. We observed higher readmission rates at 30 and 90 days, with increasing DGF duration. Compared to those without DGF, recipients with DGF lasting > 14 days had a 22–24% higher 30-day readmission rate and 27–30% higher 90-day readmission rate.

We also recognize that the need to start and continue dialysis is subjective. There can be variation regarding this decision within the center and between centers. Some centers may also be conservative with respect to dialyzing patients versus medical management. Since we have easy access to outpatient dialysis, it may result in less strict criteria for dialysis. Besides the center's practice of accepting more donors with severe AKI kidneys and long cold ischemia time, the different thresholds for dialysis may also play a role in higher DGF rates in our patient population.

Unlike previously published studies [13, 14], death-censored graft survival was not associated with the length of DGF except when DGF lasted > 28 days. Graft loss in patients with DGF lasting > 28 days was often due to a combination of donor and recipient factors. Recipient factors contributing to the graft loss identified in our study included cardiovascular complications, severe infections, acute rejection, and glomerulonephritis. For these recipients, prolonged DGF and associated outcomes appeared to be secondary to these post-transplant events.

## Conclusions

Our study is a single-center study and has several limitations. As a center that utilizes a high proportion of high KDPI, AKI, DCD, and nationally allocated kidneys, our overall incidence of DGF is higher than other centers. The study findings may not apply to centers that rely their decisions on pump pressures and higher use of machine perfusion and have shorter cold ischemia times. Our decisions to accept kidneys were not based on pump parameters, and we did not include data on machine perfusion as we mostly rely on biopsy findings.

As a result, we recognize that our experience with DGF and outcomes may be unique compared to the greater transplant community. Although we assessed hospital length of stay and readmission rates, the financial impact of DGF duration was not assessed in detail. Access to outpatient non-hospital-based hemodialysis is a practice specific to our center that has helped us decrease inpatient hospital resource utilization. This unique aspect of our practice may not be universally applicable to other centers as resource availability varies from center to center. Despite these limitations, we feel that our experience with DGF and outcomes are valuable. Despite using donors with higher risk features and overall higher rates of DGF, we have reported excellent outcomes [8, 9, 16, 19, 20]. Moreover, this current study provides granular details specific to DGF that are not available from larger database studies.

We conclude that the duration of DGF, rather than DGF itself, has greater clinical significance and is associated with kidney transplant outcomes. In this study, delayed graft function lasting up to 28 days post-transplant for most patients has no detrimental impact on graft survival. However, DGF persisting for > 28 days is associated with inferior kidney graft survival. When assessed in the context of therapeutic and timely immunosuppression, increasing duration of DGF does not increase the risk of acute rejection or progression of interstitial fibrosis. Although DGF is associated with higher readmission rates, long-term outcomes remain excellent. Future studies assessing the impact of DGF on kidney transplant outcomes should consider transitioning from the assessment of DGF as a dichotomous outcome to that of a continuum.

## Supplementary Information

**Additional file 1**: **Table S1**. Cause of death censored graft loss within 1 years. **Table S2**. Causes of death within 1 year. **Table S3** for supplement. Comparison of group with DGF < 28 days and > 28 days

## Data Availability

The datasets generated and/or analysed during the current study are not publicly available due [these are sensitive data] but are available from the corresponding author on reasonable request.

## Abbreviations

AKI: Acute Kidney Injury
AKIN: Acute Kidney Injury Network
ANOVA: Analysis of variance
ci: Chronic Interstitial Fibrosis
CIT: Cold Ischemia Time
DCD: Donation after Circulatory Death
DDKT: Deceased Donor Kidney Transplantation
DGF: Delayed Graft Function
HTN: Hypertension
IQR: Interquartile Range
KDPI: Kidney Donor Profile Index
PNF: Primary Nonfunction
UK: United Kingdom
