# Supplementary material for: Duration of delayed graft function and its impact on graft outcomes in deceased donor kidney transplantation
Source: BMC Nephrol. 2022 Apr 19;23:154. doi: 10.1186/s12882-022-02777-9 (PMC9017045; doi:10.1186/s12882-022-02777-9)
Supplement: Supplementary file 1 — Additional file 1: Table S1. Cause of death censored graft loss within 1 years. Table S2. Causes of death within 1 year. Table S3 for supplement. Comparison of group with DGF < 28 days and > 28 days [file 12882_2022_2777_MOESM1_ESM.docx]

Table 1. Cause of death censored graft loss within 1 years.

|  | **No DGF (n=696)** | **DGF <14 days (n=749)** | **DGF 15-28 days (n=217)** | **DGF > 28 days (52)** |
| --- | --- | --- | --- | --- |
| Rejection | 5 | 5 | 1 | 2 |
| Acute kidney injury and high KDPI kidney | 0 | 0 | 0 | 1 |
| Hypotension due to cardiac/cirrhosis | 0 | 0 | 0 | 3 |
| Advanced chronic changes on time-0 biopsy | 0 | 0 | 0 | 2 |
| Recurrent Glomerulonephritis | 1 | 0 | 1 | 1 |
| Thrombotic microangiopathy | 1 | 0 | 0 | 0 |
| Oxalate crystal and high KDPI | 0 | 0 | 0 | 1 |
| Infection | 3 | 1 | 1 | 0 |
| BKV | 1 | 1 | 0 | 0 |
| Graft removal during cardiac arrest | 1 | 0 | 0 | 0 |
| other | 3 | 2 | 1 | 0 |
|  | 15(2%) | 9(1.2%) | 4(1.8%) | 10(19%) |

Table 2. Causes of death within 1 year

|  | **No DGF (n=696)** | **DGF <14 days (n=749)** | **DGF 15-28 days (n=217)** | **DGF > 28 days (n=52)** | **P-value** |
| --- | --- | --- | --- | --- | --- |
| Infection | 2 | 1 | 3 | 6 |  |
| Malignancy | 1 | 1 | 0 | 0 |  |
| Cardiac/respiratory failure | 5 | 8 | 2 | 0 |  |
| Refuse to do go back on chronic dialysis | 2 | 0 | 0 | 0 |  |
| Unknown | 1 | 6 | 2 | 0 |  |
|  | 11 (1.5%) | 16(2.1%) | 7(3.2%) | 6 (11.5%) | <0.001 |

Table 3 for supplement. Comparison of group with DGF < 28 days and > 28 days

|  | DGF <28 days (n=966) | DGF > 28 days(n=52) | P value |
| --- | --- | --- | --- |
| Kidney Donor Profile Index >85 | 13% | 17% | 0.5 |
| Kidney Donor Profile Index | 53.2(25) | 62(21) | 0.013 |
| Donor age | 40.4(15) | 45.6(12) | 0.7 |
| Donor age >60 | 11% | 10% | 1 |
| Donor history of Diabetes(yes) | 7.6% | 12.8% | 0.26 |
| Donor h/o Hypertension (yes) | 29% | 44% | 0.04 |
| Donor h/o Hypertension > 10 years(yes) | 6% | 4% | 0.56 |
| Donor race African American | 8% | 4% | 0.4 |
| Donation after Circulatory Death | 26.3% | 39% | 0.08 |
| Donor Acute Kidney Injury 2 or more | 515 (53%) | 20 (39%) | 0.046 |
| Donor Acute Kidney Injury 3 | 462 (48%) | 14(27%) | 0.004 |
| Donor terminal creatinine | 3.2(2.9) | 2.6(2.2) | 0.13 |
| Cold Ischemia time >24 hours | 33% | 41% | 0.3 |
| Recipient diabetes mellitus | 48% | 54% | 0.3 |
| Recipient age >65 | 29% | 29% | 1 |
| Recipient age | 56.2(13) | 56.8(12) | 0.7 |
| Previous transplant | 9% | 10% | 0.8 |
| Male recipient | 65% | 76% | 0.14 |
| Time on dialysis | 1395.92(932) | 1540(850) | 0.3 |
| Recipient African American race | 13% | 13.5% | 0.8 |
| Donor Acute Kidney Injury Kidney Donor Profile Index>85 | 43(5%) | 4(7.6%) | 0.33 |
|  |  |  |  |
